# Analysis of Mineral and Heavy Metal Content of Some Commercial Fruit Juices by Inductively Coupled Plasma Mass Spectrometry

**DOI:** 10.1155/2013/215423

**Published:** 2013-12-18

**Authors:** Adriana Dehelean, Dana Alina Magdas

**Affiliations:** National Institute for Research and Development of Isotopic and Molecular Technologies, 65-103 Donath Street, P.O. Box 700, 400293 Cluj-Napoca 5, Romania

## Abstract

The presence of potentially toxic elements and compounds in foodstuffs is of intense public interest and thus requires rapid and accurate methods to determine the levels of these contaminants. Inductively coupled plasma mass spectrometry is a powerful tool for the determination of metals and nonmetals in fruit juices. In this study, 21 commercial fruit juices (apple, peach, apricot, orange, kiwi, pear, pineapple, and multifruit) present on Romanian market were investigated from the heavy metals and mineral content point of view by ICP-MS. Our obtained results were compared with those reported in literature and also with the maximum admissible limit in drinking water by USEPA and WHO. For Mn the obtained values exceeded the limits imposed by these international organizations. Co, Cu, Zn, As, and Cd concentrations were below the acceptable limit for drinking water for all samples while the concentrations of Ni and Pb exceeded the limits imposed by USEPA and WHO for some fruit juices. The results obtained in this study are comparable to those found in the literature.

## 1. Introduction

Fruit juices are a highly appreciated, tasty food and usually have exceptional nutritional qualities. However, they can be a potential source of toxic elements, some of them having an accumulative effect or leading to nutritional problems due to the low concentrations of essential elements, justifying the control of mineral composition in juice [1, 2].

Trace metals are present in foods in amounts below 50 ppm and have some toxicological or nutritional significance. The elements such as Na, K, Ca, and P are essential for people, while metals like Pb, Cd, Hg, and As are found to cause deleterious effects even in low levels of 10–50 ppm. However Fe, Cu, and Zn are found to be necessary in certain quantities in foods, but these elements can cause ill effects when are ingested in high amounts. Other nontoxic metals which are not harmful when present in amounts not exceeding 100 ppm include Al, B, Cr, Ni and Sn. The non-nutritive toxic metals which are known to have deleterious effects even in small quantities (below 100 ppm) are As, Sb, Cd, F, Pb, Hg, and Se [3, 4]. For this reason, the determination of both major and trace levels of metal contents in food is important for both food safety and nutritional considerations [3].

The trace element levels of fruit juices may be expected to be influenced by the nature of the fruit, the mineral composition of the soil from which it originated, the composition of the irrigation water, the weather conditions, the agricultural practices such as the types and amounts of fertilizers used, and other factors [5].

The aim of this study was the precise determination of mineral and heavy metal content from the most known commercial fruit juices present on Romanian market and to compare these results with the maximum admissible limit set in drinking water by different international organizations: United States Environmental Protection Agency (USEPA) [6] and World Health Organization (WHO) [7] and also with the values of different countries available in literature.

## 2. Experimental

21 commercial fruit juices (apple, peach, apricot, orange, kiwi, pear, pineapple, and multifruit) purchased from Romania market were investigated in this study. All determinations were carried out by the Inductively Coupled Plasma Quadrupole Mass Spectrometry. A Perkin Elmer ELAN DRC (e) was used with a Meinhart nebulizer and silica cyclonic spray chamber and continuous nebulization. The operating conditions for Perkin Elmer ELAN DRC (e) were nebulizer Gas flow rates: Nebulizer Gas flow rates: 0.92 L/min; Auxiliary Gas Flow: 1.20 L/min; Plasma Gas Flow: 15 L/min; Lens Voltage: 10.5 V; ICP RF Power: 1100 W; CeO/Ce = 0.020; Ba^++^/Ba^+^ = 0.023. The chosen conditions were a compromise between the highest ^103^Rh ion signal and the lowest percentage of doubly charge ions (obtained by the intensities ratio Ba^++^/Ba^+^, always ≤3%) and of oxide ions (obtained by the intensities ratio CeO/Ce, always ≤3%), precision better than 2% and background <30 cps.

The operating conditions were optimized daily, by using an aqueous solution containing 10 *μ*g L^−1^ of Mg, Ba, Ce, Cu, Cd, Rh, In, and Pb, and monitoring the intensities of the isotopes ^24^Mg, ^103^Rh, ^114^In, ^208^Pb, ^138^Ba, and ^140^Ce as well as the intensities at mass 69, 156 and 220 (corresponding to species ^138^Ba^2+^, ^140^Ce^16^O^+^ and background, resp.).

The majority of ICP-MS applications involve the analysis of aqueous samples, directly or following sample pretreatment, because of the advantages of working with samples in solution. In this survey, 2.5 mL of ultrapure nitric acid were added to 2.5 mL of fruit juices in a Teflon receptacle, tightly closed. Six such receptacles were inserted in a device made of six stainless steel cylinders mounted between two flanges, to confer pressure resistance. The whole system was put in an oven at 180°C for 12 hours. A colorless solution resulted, and ultrapure water was added up to 50 mL. Thus, the fruit juices samples were diluted 1 : 20 v/v.

For each sample analysis three replicates were measured in order to assure the control quality of our measurements.

## 3. Results and Discussion

The evaluation of commercial fruit juices is a important issue for consumer safety, as they are widely consumed throughout the world [8]. Determining cations, such as potassium, sodium, and calcium, in fruit juices is important due to the dietary significance of such cations. For example, recent studies have supported the contention that excess dietary sodium is a contributing factor in heart disease. Ca, though an important dietary component for most, can be an issue for patients with renal insufficiency. K is also essential for good health and is present in significant concentrations in some juices. For these reasons, accurate reporting of cation levels is helpful [9].

The obtained concentration of Na, Mg, K, and Ca for 21 commercial fruit juices are presented in Table 1. The SD of measurement samples was: 0.91 for Na, 0.17 for Mg, 3.08 for K, 0.21 for Ca.

The concentration ranges are the following for the major elements: 1.12–196.11 mg/L for Na, 13.07–140.42 mg/L for Mg, 52.52–642.34 mg/L for K and 21.54–338.35 mg/L for Ca.

The higher Ca and K content was founded in orange juices (orange 3 and orange 6), while the bigger amount of Na was detected in the multifruit juices (multifruit) and for the Mg concentration a maximum was observed in pineapple juices (pineapple).

Heavy metals composition of foods is of interest because of their essential or toxic nature. For example, Fe, Zn, Cu, Cr, Co, and Mn are essential, while As, Pb, Cd, Ni, and Hg are toxic at certain levels [10].

The concentrations of heavy metals, Cr, Mn, Co, Ni, Cu, Zn, As, Cd, and Pb, in analyzed commercial fruit juices samples are presented in Table 2. The SD of measurement juice samples was, 0.35, 3.75, 0.05, 0.17, 0.69, 3.38, 0.05, 0.03, and 0.10 for Cr, Mn, Co, Ni, Cu, Zn, As, Cd, and Pb, respectively.

The US Environmental Protection Agency (USEPA) has carried out risk assessments dealing with the toxicity by establishing References Doses (RfDs) for a large number of chemicals, including some essential trace elements. The World Health Organization (WHO) has set similar values for toxicity, termed Acceptable Daily Intakes (ADIs) [11].

We compared the heavy metals content founded in our investigated fruit juices with international organizations WHO (2008) [7], USEPA [6], recommended drinking water standards (Table 3).

Cr is an essential micronutrient for animals and plants and is considered as a biological and pollution significant element [12]. Cr in excess amounts can be toxic especially in the hexavalent form. The data obtained for most samples which were analyzed are smaller than maximum admissible limit set by WHO (2008) (50 *μ*g/L), excepting two samples: apple 3 and apricot 1. The concentrations of Cr in commercial fruit juices are bellow the USEPA (2008) maximum admissible limit of this element in drinking water (100 *μ*g/L).

In our study, the Mn concentration was above the acceptable limit for drinking water, according to USEPA standard, for all samples, but did not exceed the limit allowed by WHO (2008).

Co is a necessary cofactor for making the thyroid hormone thyroxin. Co has also been used in anemia treatment as it causes the red blood cells production. The toxicity of Co is quite low compared to that of many other metals [19]. All the samples contained Co present concentrations bellow the maximum admissible limit of USEPA (2008) and WHO (2008).

The allowed limits in drinking water for Ni are 100 *μ*g/L (by USEPA) and 70 *μ*g/L (by WHO). It can be noticed that six values of Ni concentrations from investigated fruit juices are over these limits. The higher Ni content observed for these samples could arise from contamination either from the processing step or from an existing contamination in the drinking water used in fruit juices processing.

Compared with the maximum acceptable limit for Cu concentration in drinking water, cooper's concentrations are lower than their maximum limit values.

Zn is one of the important trace elements that play a vital role in the physiological and metabolic process of many organisms. Nevertheless, higher concentrations of Zn can be toxic to the organism [25].

Cd is highly toxic and responsible for several cases of poisoning through food. Small quantities of Cd cause adverse changes in the arteries of human kidney. It replaces Zn biochemically and causes high blood pressures, kidney damage, and so forth [26]. Pb and Cd toxicity are well documented and are recognized as a major environmental health risk throughout the world [24, 27]. Because of their high toxicity, As, Pb, and Cd need to be quantified in food and beverages [28].

In this study, both concentrations of Zn, As and Cd are bellow the limits imposed by USEPA (2008) and WHO (2008).

The analysis of Pb content reveals the fact that five fruit juices samples (apple 1, apple 4, apple 5, peach 3, and orange 1) exceed the allowable limits for drinking water. Taking into account that lead is less mobile than Cd in the soil-plant system [29], the sources of fruit juices contamination are most probably either the added sugar or the water used in the fruit juices reconstruction.

The concentrations of the metals found in all fruit juices are compared with the values reported in literature for the fruit juices (Table 4).

The wide variation range of reported data in literature could be explained both by the variability of used raw materials in the fruit juices production and manufacturing processes employed. Because fruit juices are coming from different countries, the metals content reflects differences in soil composition where the fruits were grown.

## 4. Conclusions

This study presents data on the concentrations of minerals and heavy metals in commercial fruit juices (apple, peach, apricot, orange, kiwi, pear, pineapple, and multifruit) present on Romanian market. The highest content of Ca and K was found in orange juices, whereas Mg concentration had a maximum in pineapple juice. The obtained content of heavy metals and minerals in fruit juices is due to the concentration of these elements in raw materials and also is influenced by the manufacturing process. The metal concentration in raw materials depends on a number of factors, including the soil composition, the external conditions during fruit growing and fruit harvesting.

## Figures and Tables

**Table 1:** The concentrations for minerals (mg/L) of commercial juice samples.

Sample	Na	Mg	K	Ca
Apple 1	36.36	50.42	415.35	72.07
Apple 2	16.59	45.80	373.32	49.37
Apple 3	10.39	47.43	368.73	36.71
Apple 4	32.97	38.18	332.11	26.90
Apple 5	22.40	47.99	344.63	72.44
Apple 6	1.12	38.62	391.77	50.34

Peach 1	129.20	40.33	257.23	26.35
Peach 2	9.24	20.28	52.52	32.83
Peach 3	17.56	36.38	265.62	39.48

Apricot 1	100.03	30.20	301.77	24.76
Apricot 2	59.52	39.47	377.60	72.67

Orange 1	15.57	32.79	196.35	43.27
Orange 2	17.90	28.84	72.91	66.92
Orange 3	11.08	25.92	75.58	338.35
Orange 4	81.43	43.61	265.24	35.73
Orange 5	87.72	55.70	334.92	31.64
Orange 6	25.12	120.61	642.34	81.02

Kiwi	39.23	23.42	102.74	22.93

Pear	62.19	31.22	170.21	21.54

Multifruit	196.11	13.07	74.91	42.90

Pineapple	119.97	140.42	413.72	103.94

**Table 2:** The levels of heavy metals in investigated samples (*µ*g/L).

Sample	Cr	Mn	Co	Ni	Cu	Zn	As	Cd	Pb
Apple 1	4.11	213.72	0.26	18.96	99.12	180.16	3.60	0.14	24.84
Apple 2	4.00	127.08	1.24	42.46	57.66	196.96	1.52	0.24	5.86
Apple 3	55.60	268.22	8.30	58.22	104.78	379.10	<DL	0.84	4.66
Apple 4	5.69	293.16	<DL	36.22	37.58	171.24	0.94	0.26	17.40
Apple 5	23.58	342.92	4.42	123.60	64.20	103.54	4.36	1.42	75.68
Apple 6	30.90	207.12	0.74	204.40	153.68	348.76	<DL	0.8	7.82

Peach 1	39.64	229.80	7.60	26.11	403.88	568.92	1.52	0.52	5.96
Peach 2	5.61	59.60	<DL	53.12	20.72	236.94	<DL	0.64	1.94
Peach 3	30.44	198.90	6.84	25.42	322.94	1186.06	3.78	1.38	18.58

Apricot 1	55.45	244.46	7.00	35.04	139.34	445.74	1.52	0.46	5.36
Apricot 2	26.98	206.98	1.20	146.60	534.22	516.42	1.52	0.78	3.36

Orange 1	8.17	64.14	<DL	57.00	203.72	188.70	<DL	0.12	10.03
Orange 2	5.69	88.24	<DL	31.24	33.28	199.78	<DL	<DL	1.08
Orange 3	5.28	71.66	<DL	67.72	37.86	189.56	<DL	0.64	1.02
Orange 4	17.13	92.42	<DL	40.28	129.74	100.18	1.70	<DL	1.66
Orange 5	20.71	97.30	2.76	145.00	88.50	201.04	3.02	0.4	1.96
Orange 6	28.21	307.88	2.18	134.16	303.90	204.12	1.52	0.26	3.98

Kiwi	8.69	66.70	1.20	46.04	442.38	118.44	<DL	<DL	1.64

Pear	7.56	169.56	6.30	31.52	214.16	123.66	<DL	<DL	1.62

Multifruit	4.46	131.64	<DL	32.20	25.44	277.60	0.38	<DL	1.88

Pineapple	27.05	320.80	6.54	208.96	171.98	498.74	2.84	0.64	1.54

DL: detection limit, 0.001 *µ*g/L.

**Table 3:** Drinking water contaminants and maximum admissible limit by different international organizations.

	Heavy metals (*µ*g/L)								
	Cr	Mn	Co	Ni	Cu	Zn	As	Cd	Pb
US-EPA (2008)	100	50	100	100	1300	5000	10	5	15
WHO (2008)	50	400	^ a^NM	70	2000	NGL^b^	10	3	10

^a^NM: not mentioned.

^b^NGL: no guideline, because it occurs in drinking water at concentrations well below those at which toxic effects may occur.

**Table 4:** The metal concentrations of fruit juices in present study compared with the published values.

Type of fruit juices	Concentration (*μ*g/L)								Reference used method
	Cr	Zn	Cu	Co	Mn	Ni	Cd	Pb	
Orange	5.75	242–480	239–460						[13–15] GFAAS, FAAS
	11.5	842; 511	271; 309	10.3; 20	28.7				[16–19] ICP-MS, AAS
		400	400			20			[17, 20] ASS
	5.57				16–90	19.7–58.7			[17] ASS
		350.2	370.8	3.9	93; 19.6	11.3; 30			[17, 21] ICP-MS, ICP-AES
	2.4	731.5	422.3	2.8	27.5	28.3			[16] ICP-MS
	6.11	921.64; 60	515; 23	8.17; 0.9	21.56; 180	5.90; 20.5		2	[1, 22] GFAAS, ICP-MS
	5.28–28.21	100.18–204.12	33.28–303.90	2.18–2.76	64.14–307.88	31.24–145.00	0.12–0.64	1.02–10.03	Present study ICP-MS (range)

Apple		691	335; 416	30					[10, 19] RIMS
	8; 10	560; 474	535		56	13			[10, 14, 23] GFAAS, spectrofluorimetry
	16; 6.61	544.96	330.50; 73	8.39	24.42; 75	6.46; 3.9		0.2	[1, 16, 22] ICP-MS, GFAAS
	56.04	156.20	315.30	0.75		30.10	0.28–0.43	1.56–3.42	[24] ICP-MS
	4.00–55.60	103.54–379.10	37.58–153.68	0.26–8.30	127.08–342.92	36.22–204.40	0.14–1.42	4.66–75.68	Present study ICP-MS (range)

Pineapple		5730	160	3.7	1360	43.5	1.1	1.6	[1] ICP-MS
	27.05	498.74	171.98	6.54	320.80	208.96	0.64	1.54	Present study ICP-MS

Pear		370	58		140	15.3	0.3	0.7	[1] ICP-MS
	7.56	123.66	214.16	6.30	169.56	31.52	<DL	1.62	Present study ICP-MS

Peach		400	230		210	21.4	0.6	0.6	[1] ICP-MS
	5.61–30.44	236.94–1186.06	20.72–322.94	6.84–7.60	59.60–229.80	25.42–53.12	0.52–1.38	1.94–18.58	Present study ICP-MS (range)

DL: detection limit, 0.001 *μ*g/L.

## References

[1] Tormen L, Torres DP, Dittert IM, Araújo RGO, Frescura VLA, Curtius AJ (2011). Rapid assessment of metal contamination in commercial fruit juices by inductively coupled mass spectrometry after a simple dilution. *Journal of Food Composition and Analysis*.

[2] Hague T, Petroczi A, Andrew PLR, Barker J, Naughton DP (2008). Determination of metal ion content of beverages and estimation of target hazard quotients: a comparative study. *Chemistry Central Journal*.

[3] Ministry of Health and Family Welfare (2005). *Manual of Methods of Analysis of Metals. Lab. Manual 9*.

[4] Williams AB, Ayejuyo OO, Ogunyale AF (2009). Trace metal levels in fruit juices and carbonated beverages in Nigeria. *Environmental Monitoring and Assessment*.

[5] Beattie JK, Quoc TN (2000). Manganese in pineapple juices. *Food Chemistry*.

[6] http://www.cdph.ca.gov/certlic/drinkingwater/Documents/DWdocuments/EPAandCDPH-11-28-2008.pdf.

[7] WHO (2008). *Guidelines for Drinking Water Quality*.

[8] Franke SIR, Prá D, Giulian R (2006). Influence of orange juice in the levels and in the genotoxicity of iron and copper. *Food and Chemical Toxicology*.

[9] http://www.dionex.com/en-us/webdocs/88490-AB117-IC-Cations-FruitJuices-07Oct2010-LPN2605.pdf.

[10] Onianwa PC, Adetola IG, Iwegbue CMA, Ojo MF, Tella OO (1999). Trace heavy metals composition of some Nigerian beverages and food drinks. *Food Chemistry*.

[11] Goldhaber SB (2003). Trace element risk assessment: essentiality vs. toxicity. *Regulatory Toxicology and Pharmacology*.

[12] Jayana BL, Prasai T, Singh A, Yami KD (2009). Assessment of drinking water quality of madhyapur-thimi and study of antibiotic sensitivity against bacterial isolates. *Nepal Journal of Science and Technology*.

[13] López FF, Cabrera C, Lorenzo ML, López MC (2002). Aluminium content of drinking waters, fruit juices and soft drinks: contribution to dietary intake. *Science of the Total Environment*.

[14] García EM, Cabrera C, Sánchez J, Lorenzo ML, López MC (1999). Chromium levels in potable water, fruit juices and soft drinks: influence on dietary intake. *Science of the Total Environment*.

[15] Silvestre MD, Lagarda MJ, Farré R, Martínez-Costa C, Brines J (2000). Copper, iron and zinc determinations in human milk using FAAS with microwave digestion. *Food Chemistry*.

[16] Martin GJ, Fournier JB, Allain P, Mauras Y (1997). Optimization of analytical methods for origin assessment of orange juices. II. ICP-MS determination of trace and ultra-trace elements. *Analusis*.

[17] Maduabuchi JMU, Nzegwu CN, Adigba EO (2008). Iron, manganese and nickel exposure from beverages in Nigeria: a public health concern?. *Journal of Health Science*.

[18] Zhou H, Liu J (1997). The simultaneous determination of 15 toxic elements in foods by ICP-MS. *Atomic Spectroscopy*.

[19] Song K, Cha H, Park SH, Lee YI (2003). Determination of trace cobalt in fruit samples by resonance ionization mass spectrometry. *Microchemical Journal*.

[20] Eisele TA, Drake SR (2005). The partial compositional characteristics of apple juice from 175 apple varieties. *Journal of Food Composition and Analysis*.

[21] Simpkins WA, Louie H, Wu M, Harrison M, Goldberg D (2000). Trace elements in Australian orange juice and other products. *Food Chemistry*.

[22] Farid SM, Enani MA (2010). Levels of trace elements in commercial fruit juices in Jeddah, Saudi Arabia. *Medicine Journal Islamic World Academy Science*.

[23] Tabrizi AB (2007). Cloud point extraction and spectrofluorimetric determination of aluminium and zinc in foodstuffs and water samples. *Food Chemistry*.

[24] Magdas DA, Dehelean A, Puscas R (2012). Isotopic and elemental determination in some Romanian apple fruit juices. *The Scientific World Journal*.

[25] Rajkovic MB, Lacnjevac CM, Ralevic NR (2008). Identification of metals (heavy and radioactive) in drinking water by an indirect analysis method based on scale tests. *Sensors*.

[26] Rajappa B, Manjappa S, Puttaiah ET (2010). Monitoring of heavy metal concentration in groundwater of Hakinaka Taluk, India. *Contemporary Engineering Sciences*.

[27] Krejpcio Z, Sionkowski S, Bartela J (2005). Safety of fresh fruits and juices available on the polish market as determined by heavy metal residues. *Polish Journal of Environmental Studies*.

[28] Barbaste M, Medina B, Perez-Trujillo JP (2003). Analysis of arsenic, lead and cadmium in wines from the Canary Islands, Spain, by ICP/MS. *Food Additives and Contaminants*.

[29] Tudoreanu L, Prankel S, Enache L, Clarckson PA (2007). The EU metal project—metals in the environment, toxicity and assessment of limits: cadmium. *Environmental Research Advances*.

